# The application of machine learning techniques in posttraumatic stress disorder: a systematic review and meta-analysis

**DOI:** 10.1038/s41746-024-01117-5

**Published:** 2024-05-09

**Authors:** Jing Wang, Hui Ouyang, Runda Jiao, Suhui Cheng, Haiyan Zhang, Zhilei Shang, Yanpu Jia, Wenjie Yan, Lili Wu, Weizhi Liu

**Affiliations:** 1 Lab for Post-traumatic Stress Disorder, Faculty of Psychology and Mental Health, Naval Medical University, 200433 Shanghai, China; 2 The Emotion & Cognition Lab, Faculty of Psychology and Mental Health, Naval Medical University, 200433 Shanghai, China; 3 Graduate School, PLA General Hospital, 100853 Beijing, China; 4 Department of Health Care, The First Affiliated Hospital of Naval Medical University, 200433 Shanghai, China

**Keywords:** Human behaviour, Population screening

## Abstract

Posttraumatic stress disorder (PTSD) recently becomes one of the most important mental health concerns. However, no previous study has comprehensively reviewed the application of big data and machine learning (ML) techniques in PTSD. We found 873 studies meet the inclusion criteria and a total of 31 of those in a sample of 210,001 were included in quantitative analysis. ML algorithms were able to discriminate PTSD with an overall accuracy of 0.89. Pooled estimates of classification accuracy from multi-dimensional data (0.96) are higher than single data types (0.86 to 0.90). ML techniques can effectively classify PTSD and models using multi-dimensional data perform better than those using single data types. While selecting optimal combinations of data types and ML algorithms to be clinically applied at the individual level still remains a big challenge, these findings provide insights into the classification, identification, diagnosis and treatment of PTSD.

## Introduction

Posttraumatic stress disorder (PTSD) denotes a psychological disorder characterized by delayed onset and prolonged duration, resulting from exposure to or witnessing an exceptionally threatening or catastrophic traumatic incident. The primary symptoms of PTSD as the Diagnostic and Statistical Manual of Mental Disorders 5th edition (DSM-5) noted encompass intrusive experience, persistent avoidance of stimuli, negative alterations in cognitions and mood, as well as marked alterations in arousal and reactivity related to the traumatic events^1^ which can persist for extensive periods, spanning months or even years^2^. Over 70% adults encounter at least one traumatic event at some point in their lives^3^. PTSD is estimated to affect approximately 5% to 10% of the population^4^. The lifetime prevalence of PTSD varies from 1.3 to 12.2%, and the 12-month prevalence is 0.2 to 3.8% according to socio-cultural factors^5^.

Various studies have underscored the enduring detrimental impact of PTSD on an individual’s physical, mental, and social well-being, including social isolation, chronic pain, inflammation, cardiometabolic disorders, and an increased risk of chronic dementia, emphasizing the importance of identifying and predicting PTSD populations^4,6^. However, the psychopathology of PTSD involves a wide array of genetic, endocrine, demographic, and environmental factors that are not uniformly present in all individuals with PTSD, implying that more effective interventions may need to be tailored to specific groups or individuals.

In order to achieve effective interventions at the individual level, the most suitable analytical method for an individual’s unique bio-psycho-social characteristics is machine learning (ML). The concept of ML was originally coined by Arthur Samuel to signify the process of enabling computers to learn autonomously without explicit programming^7^. Traditional PTSD research approach is the top-down approach: formulating the hypothesis, designing the experiment, collecting the experimental data and finally deciding to accept or reject the hypothesis. ML methods can, on the one hand, obtain large amounts of data at a relatively low cost, and on the other hand, generalize alternative hypotheses through analyzing these data. That is, from multi-dimensional data such as text, scales, brain images, behavioral and physiological indicators, hidden information can be discovered, common features can be extracted, and the complex relationship between PTSD and different variables can be revealed. Thus, ML methods enable the bottom-up approach in PTSD research^8^.

**Fig. 1:**
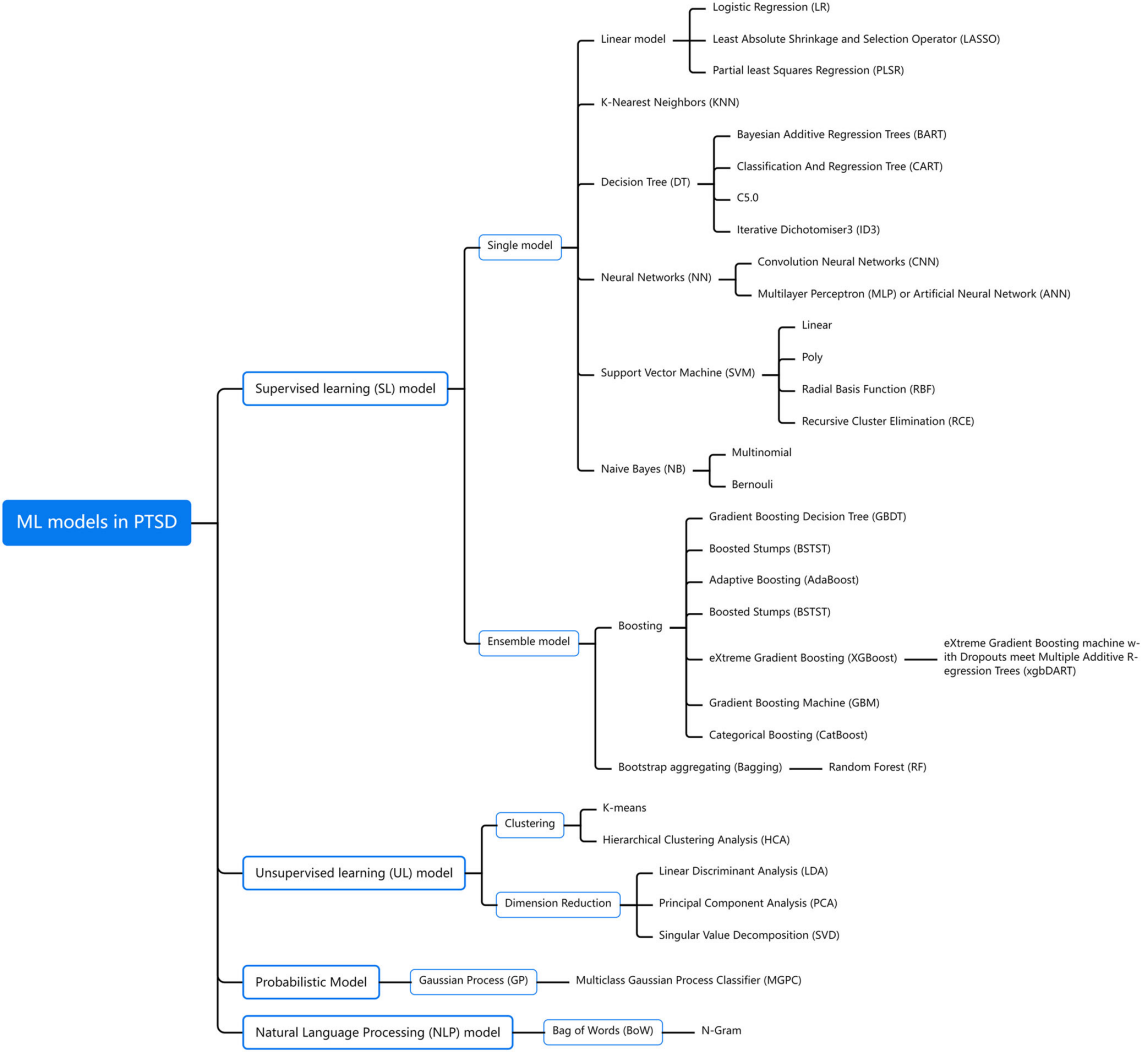
Machine learning models in posttraumatic stress disorder. The names and abbreviations of commonly used machine learning models in posttraumatic stress disorder are presented in this mind map.

ML involves a variety of algorithms, common in the field of psychology: supervised machine learning (SML), unsupervised machine learning (UML), deep learning (DL), and natural language processing (NLP). There are other algorithms such as semi-supervised learning (SSL) and reinforcement learning (RL)^9^. In PTSD, ML techniques are mainly applied in pre-diagnosis screening, identifying PTSD and its subtypes, distinguishing PTSD from other psychiatric disorders, predicting PTSD and its development trajectory, and optimizing the evaluation factors of the above four items. Previous studies have demonstrated the feasibility of using ML techniques with various data types to identify and predict PTSD, e.g. using text data to classify PTSD individuals^10^, using scales to regulate their emotion^11^ and select treatment for them^12^, using biomedical data to predict early risk^13^ and identify metabolomic-proteomic signatures associated with PTSD^14^. A classification summary of ML models commonly used in PTSD is shown in Fig. 1. In recent years, neural network architectures have been used more frequently in psychiatric studies for model-level fusion, which are highly efficient in handling high-dimensional features^15^. However, due to the low interpretability of neural network algorithms, many studies choose to use more explainable ML models, such as random forest (RF) and extreme gradient boosting (XGB), to analyze data and better reveal the contribution rate of variables^16^.

In this article, we aim to review the existing literature on the use of ML techniques in the assessment of subjects with PTSD to distinguish individuals with PTSD from other psychiatric disorder or from trauma-exposed and healthy controls or to optimize the predictors of PTSD. Furthermore, we evaluated what accuracy can ML techniques achieve in the classification of people with PTSD by analyzing different types of data and provided a quality measurement of these studies.

## Results

**Fig. 2:**
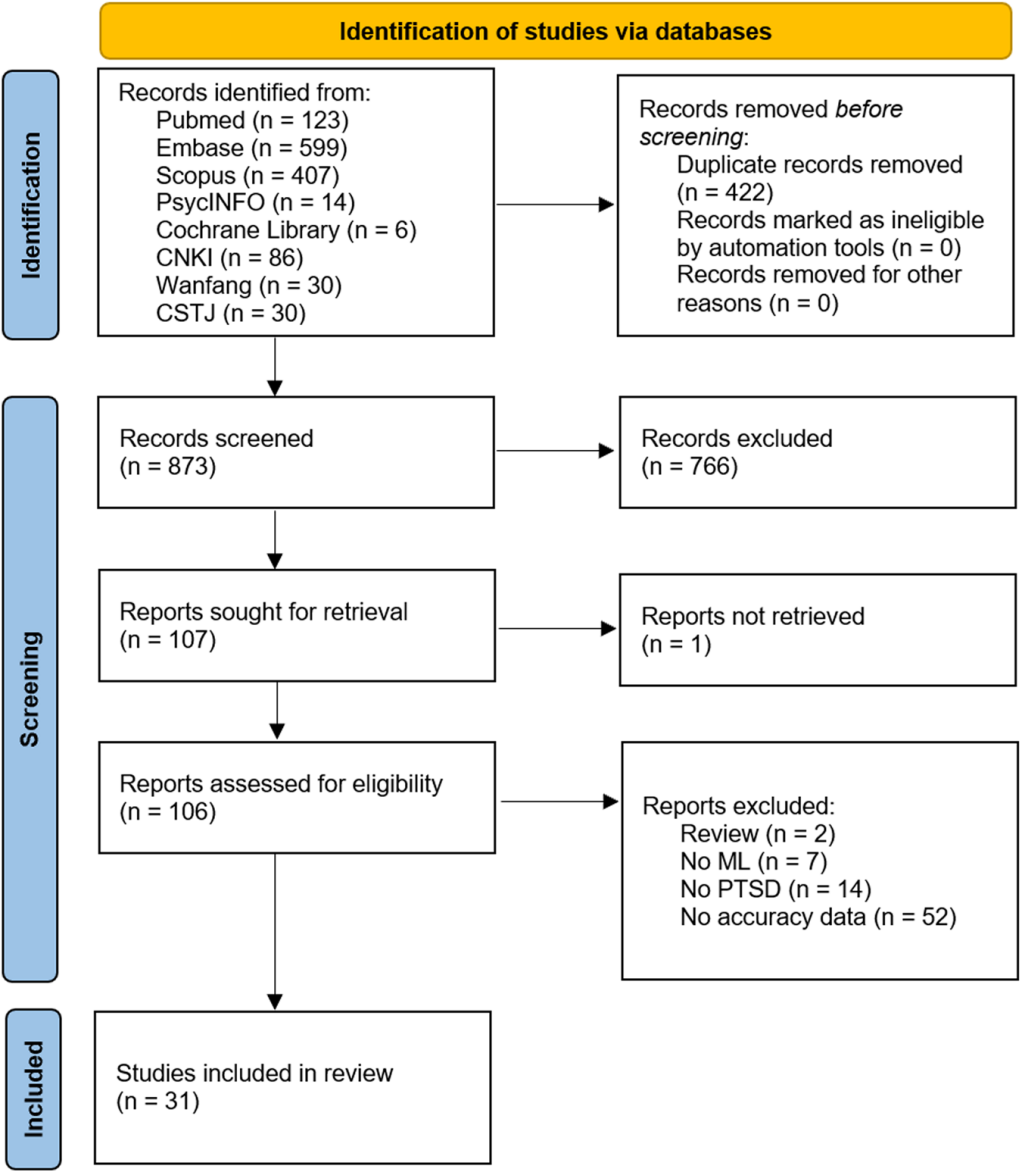
Flow diagram of review process and study selection. Note: Flow diagram according to the Preferred Reporting Items for Systematic Reviews and Meta-Analyses (PRISMA2020). *n* = number of studies/records/reports.

The initial search yielded a total of 873 unique records. After title and abstract review, 766 records were excluded. With 1 report not available for full-text, 106 publications were assessed for eligibility. After full-text review, 75 records were excluded. 7 studies investigated PTSD but did not apply an ML algorithm^17^. 14 studies used ML methods but did not apply in the field of PTSD^18^. 52 studies applied a ML algorithm to differentiate PTSD subjects from controls but did not report accuracy metrics^19^ and 2 reports^20^ are reviews. A total of 31 studies (*n* = 210,001) were included in both quantitative and qualitative synthesis (Fig. 2).

These 31 studies clearly reported what data types the ML models used and their accuracy performance metrics. 17 studies used neuroimage data^21–37^; 6 studies used scale data^38–43^; 3 studies used text data^10,44,45^; 2 studies used biomedical data^13,46^; and 3 studies used multi-dimensional data^47–49^.

### Study characteristics

The characteristics of 31 included studies are shown in Table 1. We noted an upward trend for publications in the past 4 years. 25 studies (80.6%) were published since 2018 and only 6 studies were published between 2015 and 2018 (19.4%). Most studies (74.2%; 23 of 31 articles) were conducted in the USA, China, and Canada, with 10, 7, and 6 articles, respectively. Bangladesh, Brazil, Iran, Israel, Netherlands, the Republic of Korea, Saudi Arabia and UK each contributed 1 study. A detailed comparison of regional differences in the number of publications is depicted in Supplementary Fig. 1.

**Table 1:** Characteristics of studies using machine learning techniques in posttraumatic stress disorder

First author	Data type	Diagnostic PTSD tools	Sample size	Diagnosis	ML model	Type of ML model	Accuracy	Event	Commentary
Nicholson et al. [37]	Neuroimage	CAPS-5, SCID	29	29 subjects -14 PTSD -15 HC	L1-MKL	SL	0.8000	–	
Saba et al. [21]	Neuroimage	–	28	28 subjects -14 PTSD -14 HC	LR, KNN, SVM-linear, SVM-RBF, SVM-Poly	SL	0.9920	Elder veterans from database DOD-ADNI	
Chen et al. [26]	Neuroimage	PCL-C	84	84 subjects -42 PTSD -42 HC (matched)	SVM	SL	0.7619	COVID-19 outbreak	
Li et al. [25]	Neuroimage	PCL, CAPS, SCID	46	46 subjects -23 PTSD -23 TC	RF	SL	0.6900	Earthquake children survivors	
Sheynin et al. [24]	Neuroimage	CAPS-IV	160	160 subjects -115 PTSD -45 HC (matched)	RF (with 100 estimators)	SL	0.8860	Patients in the emergency room after traumatic events	
Yang et al. [23]	Neuroimage	PCL, CAPS, SCID	86	86 subjects -33 PTSD -53 HC (matched)	FFMLNN, SVM	SL	0.7120	Earthquake children survivors	
Zhu et al. [22]	Neuroimage	PCL, CAPS, SCID	217	217 subjects -91 PTSD -126 TC (matched)	DL, SVM	SL	0.8000	Earthquakes and related traumatic events	
Harricharan et al. [30]	Neuroimage	CAPS-IV, CAPS-5	184	184 subjects -84 PTSD -49 PTSD + DS -51 HC (matched)	MGPC	SL	0.8040	Childhood trauma	
Kim et al. [29]	Neuroimage	CAPS-IV, CAPS-5, SCID	81	81 subjects -42 PTSD -39 HC	FgMDM, LDA, SVM, RF	SL	0.7309	–	
Lanka et al. [28]	Neuroimage	PCL-5	87	87 subjects -17 PTSD -42 PTSD + PCS -28 HCC (matched)	BSTST, MLP-NN, LDA	SL	0.9550	Army soldiers	Male. The actual input is based on two sets of data, so the number of people doubles and it should be 174.
Nicholson et al. [27]	Neuroimage	CAPS-IV, CAPS-5, SCID	186	186 subjects -81 PTSD -49 PTSD + DS -56 HC (matched)	MGPC	SL	0.8899	Early aversive experiences	
Nicholson et al. [33]	Neuroimage	CAPS-IV, CAPS-5, SCID	181	181 subjects -81 PTSD -49 PTSD + DS -51 HC (matched)	MGPC	SL	0.9163	Aversive experiences	
Rangaprakash et al. [32]	Neuroimage	PCL-5	87	87 subjects -17 PTSD -42 PTSD + PCS -28 HCC (matched)	RCE-SVM	SL	0.9601	Army soldiers	Male.
Salminen et al. [31]	Neuroimage	CAPS-IV	97	97 subjects -40 PTSD -57 HC	EPIC	SL	0.6900	Veterans	
Rangaprakash et al. [34]	Neuroimage	PCL-5	87	87 subjects -17 PTSD -42 PTSD + PCS -28 HCC (matched)	RCE-SVM	SL	0.9362	Army soldiers	Male.
Jin et al. [35]	Neuroimage	clinical	149	149 subjects -73 PTSD -76 HC (matched)	RCE-SVM	SL	0.9420	Earthquake survivors	
Liu et al. [36]	Neuroimage	CAPS-IV	40	40 subjects-20 PTSD -20 HC (matched)	SVM	SL	0.9250	Motor vehicle accident	
Ramos-Lima et al. [38]	Scales	CAPS-5	112	112 subjects	C5.0 (DT), GBM, Elastic Net, RF, RBF-SVM	SL	0.9020	Half were victims of sexual assault	
Jiang et al. [39]	Scales	SCID-5	1265	1265 subjects	RF	SL	0.9500	Veterans	
Ge et al. [40]	Scales	CRIES	2099	2099 subjects -803 PTSD -1296 HC	XGBoost	SL	0.7550	Earthquake children survivors	
Leightley et al. [41]	Scales	PCL-C	13690	13690 subjects -541 PTSD -13149 HC	RF, SVM, ANN, Bagging	SL	0.9700	Air force soldiers	
Wshah et al. [42]	Scales	PCL-5	90	90 subjects	SVM, NB, LR, RF, 2 ensemble methods (hard voting, soft voting)	SL	0.8799	Criterion A traumatic events	Data derived from smartphone apps.
Magoc et al. [43]	Scales	PCL-C	740	740 subjects	NN, NB, DT	SL	0.9176	Firefighters	
Shahid et al. [47]	Multi-dimensional	MINI 5.0.0, a self-report form of the Bengali translated questionnaire	164	164 subjects	RF, CNN	SL	0.9920	Refugees (*n* = 44), slum-dwellers (*n* = 35), and engineering students (*n* = 85)	Sketches, EEG.
Worthington et al. [48]	Multi-dimensional	AUDADIS-IV	34653	34653 subjects -2785 PTSD -31868 HC	GBM, classification trees, penalized logistic regression, and BART	SL	0.9509	Alcohol related events	Demographic information, individual trauma exposure, social support, psychopathology, publicly-available data about crime rates, educational attainment, employment rates, and regional economies.
Tahmasian et al. [49]	Multi-dimensional	CAPS-version 1, SCID	64	64 subjects -32 PTSD -32 HC (matched)	SVM	SL	0.9160	War-related events	Scales, EEG.
Sawalha et al. [10]	Text	PCL-C	275	275 subjects -87 PTSD -188 HC	a super learner (combines RF, GB, LDA, SVM)	SL	0.8040	Veterans and other civilians	Recordings (audio and visual) and transcripts from semi-structured clinical interviews.
Zafari et al. [44]	Text	chart review and ICD-9 diagnoses code for PTSD	154118	56795 subjects -195 PTSD -56600 non-PTSD	RF, CNN, MLNN, Word Embedding, BoW	SL, UL	0.9900	–	Negative instances were randomly selected according to the number required by the ML models (totally 154118 patients).
He et al. [45]	Text	CAPS-IV, SCID	300	300 subjects -150 PTSD -150 HC	DT, NB, SVM, PSM, n-gram	SL, UL	0.8200	Child abuse, sexual abuse, traffic accident, war, domestic violence, death of a loved one, robbery, and fire	Self-narratives and psychiatric diagnoses.
Lekkas et al. [46]	Biomedical	SCID	185	185 subjects -150 PTSD -35 TC	xgbDART, PLS, LASSO regularized GLM, SVM-RBF, RF	SL	0.7710	Childhood abuse	Data derived passively from a smartphone app.
Schultebraucks et al. [13]	Biomedical	CAPS-IV, IES-R	417	273 subjects -12 PTSD -261 non-PTSD	XGBoost	SL	0.9730	Serious injury	At T5, CAPS-IV interview data were available for N = 273 participants, while the total sample size is 417.

*HC* healthy controls, *HCC* healthy combat controls, *TC* trauma-exposed controls.

*Diagnostic instruments:* *AUDADIS-IV* Alcohol Use Disorder and Associated Disability Interview Schedule, DSM-IV Version, *CAPS* Clinician-administered PTSD Scale, *CRIES* Children’s Revised Impact of Event Scale, *ICD* International Classification of Diseases, *IES-R* Impact of Event Scale-Revised, *PCL* Posttraumatic Checklist, *PCL-C* Posttraumatic Checklist, Civilian Version, *SCID* Structured Clinical Interview for DSM–IV.

*Machine learning techniques:* *ANN* Artificial Neural Network, *Bagging* Bootstrap aggregating, *BART* Bayesian Additive Regression Trees, *BoW* Bag of Words, *BSTST* Boosted Stumps, *CNN* Convolution Neural Networks, *DT* Decision Tree, *EPIC* evolving partitions to improve classification, *FFMLNN* Feedforward Multi-Layer Neural Network, *FgMDM* Fisher geodesic Minimum Distance to the Mean, *GB* Gradient Boosting, *GBM* Gradient Boosting Machine, *GLM* Generalized Linear Model, *KNN* k-nearest Neighbors, *LASSO* Least Absolute Shrinkage and Selection Operator, *LDA* Linear Discriminant Analysis, *LR* Logistic Regression, *MGPC* Multiclass Gaussian Process Classifier, *MKL* Multiple Kernel Learning, *MLNN* Multi-Layered Neural Network, *MLP-NN* Multilayer Perceptron Neural Network, *NB* Naive Bayes, *PLS* Partial Least Squares, *PSM* Product Score Model, *RBF* Radial Basis Function, *RCE-SVM* Recursive Cluster Elimination Support Vector Machine, *RF* Random Forest, *SL* Supervised Learning, *SVM* Support Vector Machine, *xgbDART* eXtreme Gradient Boosting machine with Dropouts meet Multiple Additive Regression Trees, *XGBoost* eXtreme Gradient Boosting, *UL* Unsupervised Learning.

### Emerging patterns in the utilization of ML techniques

Among the including studies, those employed SML algorithms follow a consistent approach that is training the ML models in a labeled dataset, iteratively assessing, contrasting, and selecting variables that can effectively discriminate between PTSD and non-PTSD cases, in order to achieve optimal accuracy on an unlabeled test dataset. In contrast, UML models are trained in an unlabeled dataset to cluster individuals and ascertain pertinent latent factors. A pair of studies employed an unsupervised learning approach to construct latent profiles utilizing data from electronic medical records^44^ or self-narratives alongside psychiatric diagnoses^45^ and then constructed a comparative analysis of patient characteristics among the derived profiles to identify distinctive features for classification purposes. Illustrations of frequently employed linear algorithms in the literature under investigation encompass linear kernel-based support vector machines (SVM)^21^, extreme gradient boosting (XGBoost)^13,40^, logistic regression (LR)^42^, elastic net (EN)^38^ and least absolute shrinkage and selection operator (LASSO) regularized generalized linear model (GLM)^46^. Illustrations of frequently employed non-linear algorithms include radial basis function kernel-based SVM (RBF-SVM)^21,46^, alternating or hierarchical multi-label decision trees (DT)^38,43,45^, and multi-layer perceptron artificial neural networks (MLP-ANN)^23,28,44^. In some cases, supervised learning (SL) algorithms were integrated with unsupervised dimension reduction and clustering algorithms to effectively extract and discern significant features from the data. Some examples of these techniques include autoencoder and representation learning, e.g. bag of words (BoW)^44^, n-gram^45^, and recursive cluster elimination support vector machines (RCE-SVM)^32,34,35^.

### Meta-analysis of classification accuracy proportions and assessment of publication bias

**Fig. 3:**
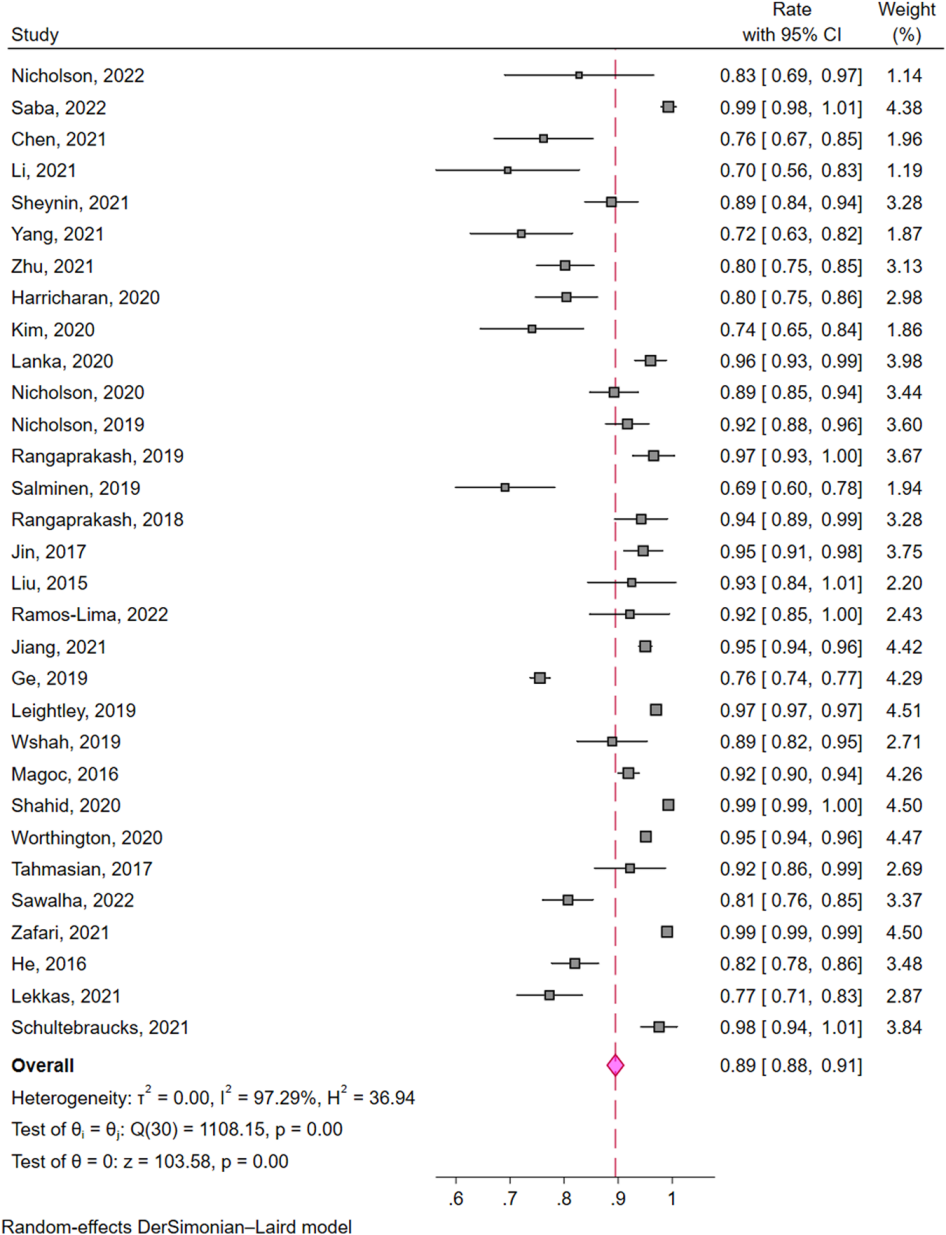
Forest plot of studies applied machine learning in posttraumatic stress disorder. Rate means the effect size in the meta-analysis, which is accuracy. 95% CI means 95% credible interval. Weight of a study is calculated by weight = 1/(se^2^ + *t*^2^). In this formula, “se” is the standard error of its accuracy, and “*t*” is a variable responding the level of the study based on the *Q* test and its effect size (i.e., accuracy).

**Fig. 4:**
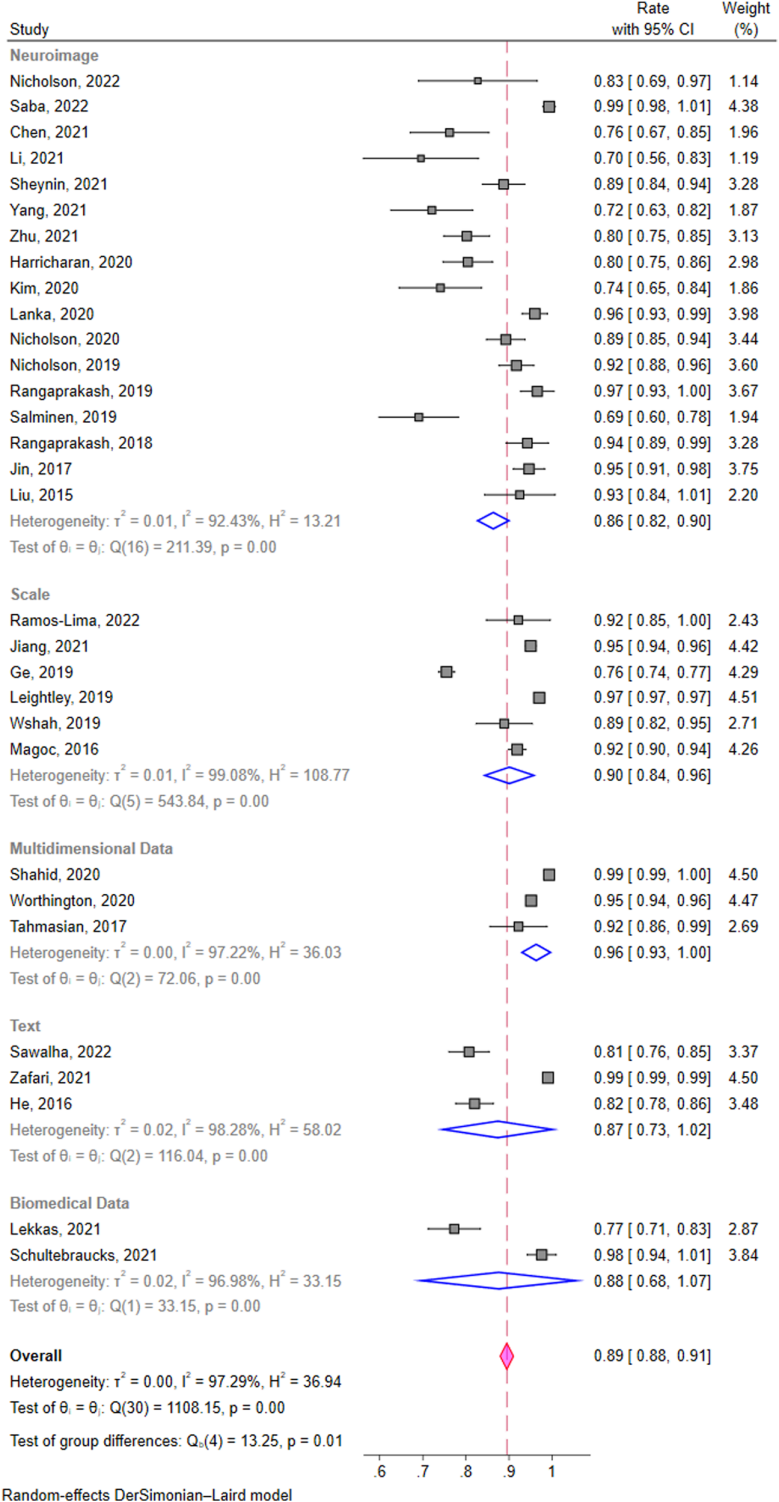
Subgroup analysis of studies applied machine learning in posttraumatic stress disorder grouped by data types. Rate means the effect size in the meta-analysis, which is accuracy. 95% CI means 95% credible interval. Weight of a study is calculated by weight = 1/(se^2^ + *t*^2^). In this formula, “se” is the standard error of its accuracy, and “*t*” is a variable responding the level of the study based on the *Q* test and its effect size (i.e., accuracy). The pooled estimate of each subgroup is shown right below the studies in that group.

**Fig. 5:**
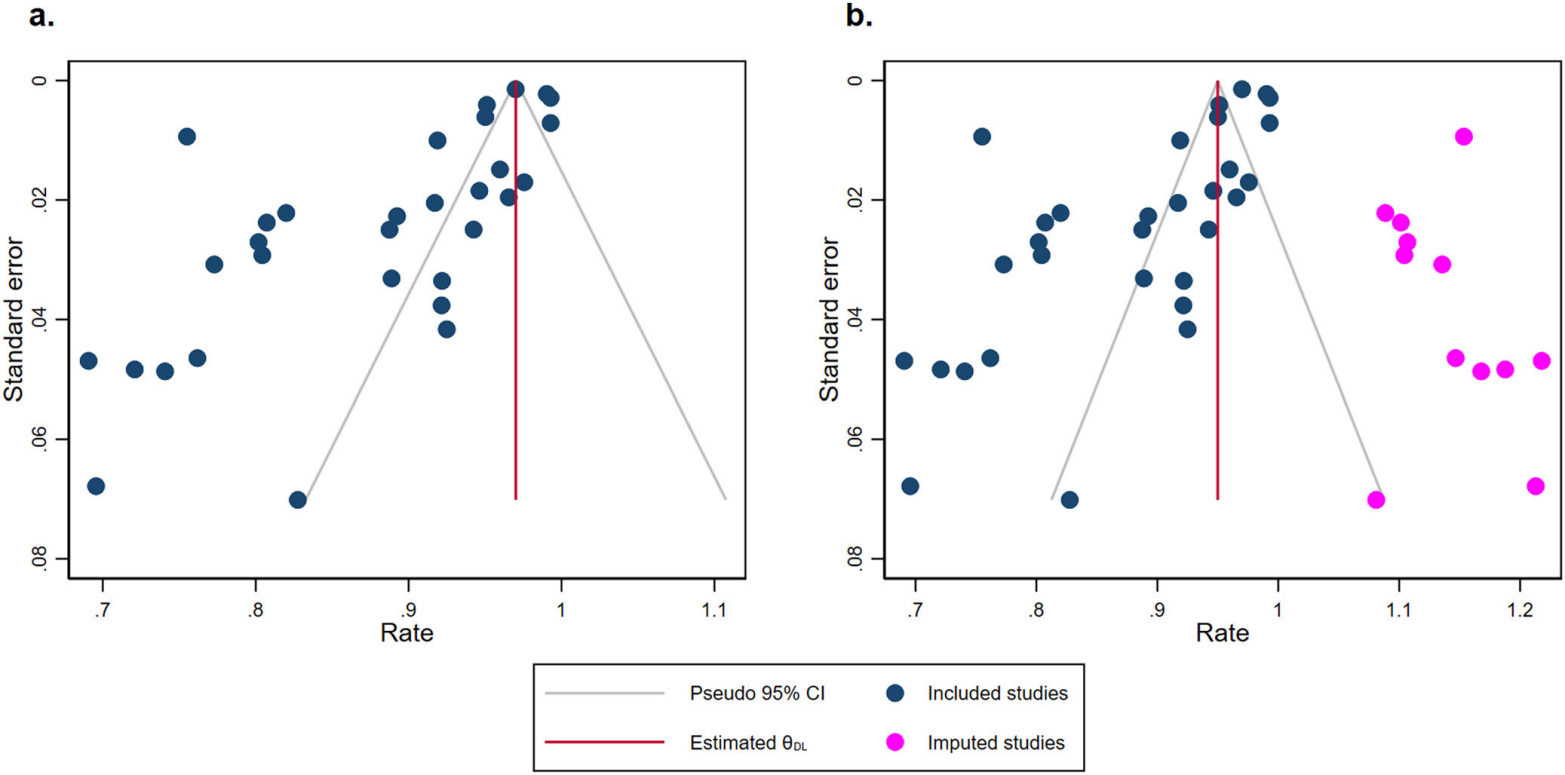
Funnel plot of studies. Note: The visually asymmetric funnel plot before trim and fill (**a**) were the evidence of publication bias. 12 studies were added after trim and fill (**b**). The gray funnel line represents the 95% confidence interval. The red line represents the estimated effect size. The dark blue dots represent the included studies’ effect sizes. The magenta dots represent the imputed studies’ effect sizes.

All of the 31 studies (*n* = 210,001) were included in the calculation of pooled estimates of classification accuracy. The overall accuracy of classification models devised by ML algorithms was 0.89 (95% confidence interval (CI) of [0.88, 0.91]) (Fig. 3). We identified 17 studies using neuroimage data to classify PTSD patients (pooled estimates [95%CI] = 0.86 [0.82, 0.90]), 6 studies using scales data (pooled estimates [95%CI] = 0.90 [0.84, 0.96]), 3 studies using text data (pooled estimates [95%CI] = 0.87 [0.73, 1.02]) and 2 using biomedical data (pooled estimates [95%CI] = 0.88 [0.68, 1.07]). Pooled estimate of ML models’ classification accuracy of 3 studies using multi-dimensional data is 0.96 ([95%CI] = [0.93, 1.00]) which is the highest among those obtained from other single data types. The estimates of accuracies are significantly different among subgroups (*p* ≤ 0.01) (Fig. 4). All the Q-values with p less than 0.05 in Fig. 3 and Fig. 4 suggested a significant heterogeneity across studies both in and among subgroups. Galbraith plot also revealed several dots outside the 95%CI lines indicating heterogeneity between studies (Supplementary Fig. 2). The sensitivity analysis carried out leave one out analysis by omitting each study demonstrating that no individual study affected the robust results from meta-analysis (Supplementary Fig. 3). Evidences of publication bias were the visually asymmetric funnel plot and the result of Egger’s test (*p* ≤ 0.01). After trim and fill adding 12 studies, the overall pooled classification accuracy proportion increased from 0.89 (95% CI [0.88, 0.91]) to 0.95 (95% CI [0.93, 0.97]) (Fig. 5).

### Quality assessment

As shown in Supplementary Table 2, we evaluated the methodological features of the included studies regarding the important points to be considered in machine-learning-based studies^50^. According to the assessment tool proposed by L.F. Ramos-Lima et al.^51^ (Supplementary Table 1), approximately 41.9% (13 out of 31 articles) of the articles included in this review employed a statistical sample or a larger sample that accurately represented the target population of the study. A majority of the studies (51.6%; 16 out of 31 articles) implemented methodologies to mitigate the influence of confounding variables, such as age, gender, or trauma type. In 64.5% of the studies (20 out of 31 articles), a rigorous and unbiased assessment conducted by independent evaluators was employed to quantify the presence of PTSD symptoms. The remaining studies utilized self-report measures, medical records, or non-blinded interviews to gather data. Additionally, a vast majority of the studies (96.8%; 30 out of 31 articles) provided comprehensive details regarding the ML techniques employed, while all of the studies (100.0%; 31 out of 31 articles) explicitly specified performance metrics such as accuracy, area under the curve (AUC), sensitivity, and specificity. A subset of the studies (25.8%; 8 out of 31 articles) presented details regarding the presence of missing data and the approach employed to address them, predominantly through imputation methods. This aspect is significant in the context of ML implementation and holds the potential to impact the outcomes based on the chosen technique. A substantial majority of the studies (93.5%; 29 out of 31 articles) employed an independent and distinct dataset to assess the performance of the generated model. Furthermore, a notable proportion of the studies (45.2%; 14 out of 31 articles) provided an account of their strategies in managing the class imbalance issue, which holds significance in calibrating and comprehending accuracy metrics. It is worth noting that a significant number of these studies maintained an equivalent sample size for both cases and controls. A comprehensive description of techniques employed for feature selection and/or hyperparameter optimization was provided in the majority of the studies (93.5%; 29 out of 31). These techniques are essential for reducing dimensionality and fostering the development of more resilient and streamlined models.

## Discussion

This systematic meta-analysis evaluated 31 studies used ML techniques to assess PTSD involving totally 210,001 participants in the calculation and yielded an overall estimate classification accuracy of PTSD of 89.4%. The majority of the studies (80.6%, 25 of 31 articles) applying these new methods in PTSD have been published in recent five years, indicating that the related computer technology has been increasingly mature and accessible.

The forest plot (Fig. 3), subgroup analysis (Fig. 4) and Galbraith plot (Supplementary Fig. 2) revealed the heterogeneity between both studies and subgroups and accuracies varied significantly. Heterogeneity between subgroups may stems from differences in data types, resulting in different methods of data collection, different levels of difficulty in sample selection, and different applicable machine learning algorithms. Additionally, different PTSD assessment tools, demographic characteristics of samples, and diverse study design are all possible sources of heterogeneity between studies. In our review, the sample size of included studies varied wildly ranging from 28 to 154,118. The effect size in the meta-analysis of this study is accuracy. Although the sample size is of concern, more attention is paid to the ratio of positive ones to the total. It determines both the effect size and the weight of studies in single proportion meta-analysis. Therefore, changes in sample size alone have little effect on the result. Its robustness was also confirmed by the sensitivity analysis (Supplementary Fig. 3). But small sample size sacrifices ML model performance which can be a source of heterogeneity.

The funnel plot (Fig. 5) was clearly asymmetrical, suggesting publication bias in the studies. This may be due to the fact that researchers tend to use models that perform well and choose to report the optimal model and its accuracy metrics.

The result of quality assessment is also noteworthy. The number of studies in the two subgroups: neuroimage and scales was relatively large, and the consequence revealed some commonalities. The process of collecting neuroimage data is time-consuming, laborious, and costly. As a result, the sample size is generally small and underrepresented. However, the collection process is carried out by professionals, which allows for better control of confounding variables and is less prone to missing data. The scale data collection is relatively simple, and a large sample size can be obtained easily. But the collection process is difficult to control by the experimenter, the confounding variables are not easy to control, and there may be a significant difference in the number of samples between PTSD and non-PTSD. Multi-dimensional data is closer to the real situation, but the problems in the process of collecting various single types of data are also concentrated, resulting in higher integrated and more complex ML algorithms and better performance.

Our study is one of the few existing systematic reviews of using ML techniques in trauma-related disorders and to conduct a meta-analysis on this topic. It quantitatively proves the effectiveness of ML technology in the field of PTSD, and provides AI-empowered (AI, artificial intelligence) evidence and ideas for the screening, diagnosis, treatment, and prognosis of PTSD. At the same time, our study learns from the published systematic reviews in this field^51^, reveals the limitations of the evidence through quantitative analysis, points out the advantages and disadvantages of ML models when using various types of data, and set some lights on the direction for better application in PTSD in the future. DL is the current trend of ML development, and it is rarely used in existing PTSD research. Its excellent processing ability for high-latitude data will play a prominent role in future PTSD research^52^.

In recent years, AI has been increasingly applied and contributing in clinical practice. Will high-performance ML models threaten the position of psychiatrists^53^? Never. Human experts play an important role in AI+mental health. First of all, as the title suggests, ML is a technique, and the psychiatrist is the diagnostic. In Chinese, ML is “Shu” and psychiatrists are “Tao”. Their relationship is the same as method and theory. Psychiatrists make diagnoses based on the data provided by ML models who are not qualified to make. Second, ML cannot fully guarantee the quality of information. Since ML models are mostly networked, the information they collect is uneven, and human experts need to check it, so that ML models can become a reliable source of mental health information. Thirdly, there are legal risks in using ML models. It may cause patients’ privacy leakage on the one hand, and ethical issues such as misdiagnosis on the other hand without the quality to bear the corresponding legal responsibility. So human experts need to make legal guarantees^54^. Fourth, the ML model provides judgment based on the existing program, and can only be learned and judged according to the original program if the parameters are not changed. In the face of sudden new situations, the ML model does not have the ability to adjust in time, but will lower the original performance because of the emergence of this special sample. At this point, human experts can play a role in correcting deviations, thus helping the model learn new samples.

Pooled estimates of classification accuracy from multi-dimensional data (0.96 [0.93, 1.00]) are the highest comparing to those obtained from other single data types (0.86 [0.82, 0.90] to 0.90 [0.84, 0.96]). Similar results have been found in related fields such as the diagnosis and treatment of other mental disorders. For example, Lee Yena et al. had found that predictive ML models using multiple data types reached highest overall classification accuracy of 0.93 in predicting the therapeutic outcomes of depression while the models with lower-dimension data reached the proportion of 0.68-0.85^55^. Katharina Schultebraucks et al. utilized a neural network approach to prove that the integration of multi-dimensional data provides a stronger prediction of both PTSD and major depressive disorder (MDD) than one source independently with an area under curve (AUC) of 0.90 and 0.86 respectively^52,56^. However, the standardization in ML models reports needs to be considered so as to provide some inspirations into the consistency and comparability of the results. First, what are the architecture of the ML models? Are they single or ensembled? Supervised or unsupervised? The models in comparison should be of the same type. Second, the performance metrics of the model should be reported for different needs. For example, sensitivity should be reported for the ability to screen positive patients, specificity should be reported for the ability to screen healthy people, and the Youden index should be reported for the optimal cut-off of a questionnaire. Third, the performance evaluation methods of the model should be flexible. While accuracy and AUC are common indicators to evaluate a model, in practical application, the generalization, complexity, operability, time cost, hardware cost and other factors of the model should be considered.

ML is an effective method applied to PTSD recognition and prediction. This data-driven approach can reduce the gap between experienced and inexperienced clinicians and therefore might eliminate a proportion of the reliance on experienced clinicians in the assessment of PTSD. But there’s still a long way to go from the lab to the hospital. First, most of the existing studies use symptomatic scales of PTSD, such as PCL-5^57^, as the criterion to identify PTSD. However, the scale of subjective reporting is not accurate, and there are phenomena such as recall bias, concealment, and fatigue effect, which can lead to misreporting, omission, and false positive. Second, PTSD has a small clinical diagnosis and is often comorbid with other psychiatric disorders^58^, such as depression, anxiety, and substance abuse, resulting in a smaller positive sample available for scientific research. Third, there are gender differences in the prevalence of PTSD^59^. The available data suggest that PTSD occurs twice in women than men^4^. This means that the same is true in the data that can be learned by the ML model. As a result, the model learns the characteristics of female patients more thoroughly, the better it diagnoses possible female patients. For men, the reverse is true. The less data can be learned from male patients, the less likely it is to be diagnosed in the future. This may lead to more severe gender disparities. Fourth, influenced by cultural differences, Easterners are more introverted and Westerners are more extroverted. Existing data may lead ML models to provide similar results of gender differences. The same can happen with the localization of symptomatic scales and public health emergencies^60^. As a result, the diagnosis of PTSD has become a process that varies from time to time and from place to place and potential limitations occur in the overall prevalence. Although the DSM-5 is now the globally accepted standard, the diagnosis of PTSD cannot be generalized when it comes to the specific individual and his unique life experience in a hospital office. Therefore, ML models still need to learn a lot of knowledge, such as gender differences, cultural differences, comorbidities, public health emergencies, etc.

And our findings also implicate that the data collected from web-based methods (such as smartphone apps^42^ or Global Positioning System (GPS) data derived passively from a smartphone^46^) can be used for PTSD which brings great convenience to clinical practice. In the actual collection of data, it is inevitable to use these efficient collection methods, which will involve personal privacy, social ethics, and potential selection bias^61^. How to improve the rigor of the procedure, the protection of private information, the avoidance of social discriminatory bias, and the iatrogenic self-fulfilling prediction will be the problems that need to be solved gradually in the future^62^.

All of the included studies used SML algorithms and only two of them used unsupervised methods. SL exhibits robust capability in understanding data characteristics when provided with labeled data, enabling accurate predictions and classifications. Conversely, while UL finds numerous applications, its learning efficacy might be impacted in situations where data labeling is insufficient. Therefore, SML algorithms are prevalently favored and frequently employed for the diagnosis and detection of various disorders. Currently, for reviews both about other mental disorders and in non-psychological fields, this is the case in most of them. In a study about therapeutic outcomes in depression, twenty-four out of the total twenty-six included articles (92%) reported the utilization of SML methods^55^. In a systematic review regarding trauma-related disorders, only three out of the forty-nine included studies used UML techniques and the others (94%) all used SML techniques^51^. A review focusing on the effectiveness of ML techniques on voice disorders only considered SML algorithms because they are more commonly used in diagnosing and screening disorders^63^. In an article about artificial intelligence in identifying left ventricular scar using cardiac magnetic resonance imaging, thirty-one of the total thirty-five studies (89%) used supervised methods and significantly outperformed in sensitivity and specificity than unsupervised models^64^. UML models are commonly used for clustering and dimensionality reduction. The advantages are that the data does not need to be labeled, which avoids the difficulty of establishing PTSD diagnostic standards and effectively saves costs. Moreover, UML can be applied for anomaly detection and pattern recognition, helping to better discover the hidden characteristics, patterns and anomalies in the population. UML is also one of the paradigms that DL has been continuously exploring and innovating in recent years. Deep neural networks (DNN) use structures and methods like autoencoders represent and generate high-dimensional and unstructured data effectively (such as images, text, speech, etc.). However, the UML methods have the disadvantage of poor interpretability, and it is difficult to explain and convincing when used in the diagnosis of PTSD. At the same time, due to the lack of labels and objective functions, it is difficult for models to evaluate and adjust parameters^65^.

## Methods

We conducted a meta-analytic and systematic review following the reporting checklist in the Preferred Reporting Items for Systematic Reviews and Meta-Analyses Statement (PRISMA2020)^66^ (Supplementary Table 3). The protocol for this review was pre-registered at PROSPERO (CRD42023342042)^67^. We assess existing research endeavors that have employed ML algorithms to discern biological and phenomenological attributes with the potential to ascertain, diagnose, and prognosticate PTSD. Within the context of the meta-analysis, we compute pooled estimations of classification accuracy for ML-derived models, i.e., the proportion of correctly (vs. incorrectly) classified PTSD cases by different types of data. All analyses were based on previous published studies and thus no ethical approval and patient consent are required.

### Search strategy

We searched Pubmed, Embase, Scopus, PsycINFO and Cochrane Library for publications in English. Publications in Chinese were searched through China National Knowledge Infrastructure Database (CNKI), Wanfang database, and China Science and Technology Journal Database (CSTJ) from 2000 to May 2022. We used the union set of entry terms and Medical Subject Headings (MeSH) terms as text words to search in titles and abstracts and every text word was searched as a whole. The search terms for machine learning were: ((“Machine Learning”) OR (“Deep Learning”) OR (“Hierarchical Learning”) OR (“Support Vector Machine”) OR (“SVM”) OR (“Reinforcement Learning”) OR (“Natural Language Processing”) OR (“Semi-supervised Learning”) OR (“Gaussian process”) OR (“Cross-validation”) OR (“Cross Validation”) OR (“Regularized Logistic”) OR (“Linear Discriminant Analysis”) OR (“LDA”) OR (“Random Forest”) OR (“Naïve Bayes”) OR (“Naive Bayes”) OR (“Bayesian”) OR (“Least Absolute Shrinkage And Selection Operator”) OR (“LASSO”) OR (“Elastic net”) OR (“RVM”) OR (“Relevance Vector Machine”) OR (“Pattern Recognition”) OR (“Pattern Classification”) OR (“Computational Intelligence*”) OR (“Machine Intelligence”) OR (“Knowledge Representation*”) OR (“Big Data”) OR (“Artificial Intelligence”)) AND ((“PTSD”) OR (“Post-traumatic Stress Disorder*”) OR (“Posttraumatic Stress Disorder*”) OR (“Post Traumatic Stress Disorder*”) OR (“Stress Disorder*, Post Traumatic”) OR (“Stress Disorder*, Posttraumatic”) OR (“Stress Disorder*, Post-traumatic”)). See Supplementary Data 1 for detailed information on the search terms. A comprehensive search for relevant literature will also be conducted, which will entail tracing the references of the included studies to identify potential eligible sources.

### Inclusion and exclusion criteria

This review encompassed all peer-reviewed publications that investigate the impact of ML methodologies on clinical practice for PTSD. All identified publications underwent a rigorous screening process, wherein the titles and abstracts were assessed for eligibility. The inclusion criteria were deliberately broad in order to enhance the search sensitivity. The specific inclusion criteria are summarized as follows: (1) PTSD was the dependent variable in the study. (2) Data related to the use of ML methods for PTSD are reported. (3) There is a clear type of data processing such as text, neuroimage, scale, biomedicine and multi-dimensional types. (4) Accuracy (ACC) metrics of ML algorithms are reported.

Studies were excluded if one of the following conditions is met: (1) The study is one of the following types: qualitative studies, editorials, letters, case studies, comments, notes, reviews, protocols or meta-analyses. (2) There is an explicit reference to non-PTSD related brain injury and genetic and biological studies. (3) No ML methods are used to study PTSD. (4) PTSD is not the dependent variable in the study. (5) The study does not report ML model’s accuracy metrics. (6) The study is not in Chinese or English.

In the meta-analytic quantitative synthesis, only peer-reviewed publications meeting the following criteria were included: reporting a measure of classification accuracy and providing information on the sample size of the analysis set. Non-refereed publications were excluded from the meta-analytic synthesis.

### Data extraction

The titles and abstracts of the identified articles were independently screened by two researchers (JW and HO). Subsequently, they acquired and thoroughly examined the full texts of potentially relevant articles. In cases of disagreement, RJ acted as the final arbiter for decision-making. Throughout the primary and secondary screening processes, all activities were overseen by WY and LW to ensure quality control. Data extracted from the articles encompass four key domains: (1) study characteristics (authors, year of publication, data type, diagnostic PTSD tool); (2) participant information (count type, sample size, diagnosis); (3) models (ML model, accuracy, other measures). (4) Additional information deemed pertinent to the study, including event details and accompanying commentary have also been extracted. Requests for supplementary information were made to the authors of two studies, with one author promptly responding and providing the requested data. SC and HZ assisted in the interpretation of the findings. Subsequently, all authors engaged in comprehensive discussions regarding the results and made significant contributions to the final version of the manuscript.

In cases where multiple measures of classification performance metrics were reported in the studies, all measures were initially extracted. Subsequently, the accuracies were either extracted directly or calculated, and these values were utilized in the quantitative analyses. If a study reported results from multiple ML models (e.g., using different ML methods, sets of features), the classification accuracy of the best performing model used in the hold-out set or the balanced accuracy was included in the calculation of pooled estimates.

### Quality analysis

We conducted a quality assessment using the tool which Luis Francisco Ramos-Lima proposed in 2020^51^, as there was no such instrument in ML studies before that. For studies using ML techniques in healthcare, the specific dimensions of quality analysis are described in Supplementary Table 1. It is divided into nine domains relating to the sample quality and effect size of a study. The first three dimensions are sample representativeness, confounding variables, and outcome assessments which are the most concerned aspects of individuals in healthcare researches implement with ML techniques. The remaining six dimensions can evaluate the quality and affect the performance of a ML approach: the model description, its accuracy or other performance metrics, how missing data and class imbalance problems had been handled, whether the model had been tested on unseen data, and whether the results were optimized using hyperparameter optimization or feature selection procedures. This review does not include studies that are of low quality or clearly have design and method flaws. The results are represented in the Results Section and shown in Supplementary Table 2. ZS and YJ independently conducted quality assessments of the risk of bias for the studies included in this review. In the event of discrepancies between the two reviewers, WL consulted to resolve any disagreements.

### Data synthesis and meta-analytic procedures

All analyses were based on previous published studies and thus no ethical approval and patient consent are required. We extracted both qualitative and quantitative data as mentioned above from each selected study. Then, we conducted meta-analysis of studies where data availability allows summary estimation for accuracy of 95% confidence intervals. For each included research, the precise count of accurately classified occurrences was ascertained by multiplying the documented proportion of classification accuracy by the sample size (n) and rounding the resulting product to the nearest integer value. Forest plots were used for the presentation of results. A random effects model was applied to estimate the overall accuracy of all the included studies. All statistics were calculated in STATA MP 17 with 0.05 as the significance level.

We chose the Q-value as an indicator of heterogeneity, with p less than 0.05 suggesting a significant heterogeneity across studies. Funnel plots and Egger’s test^68^ were adopted to detect publication bias for there were more than 10 studies reporting the primary outcomes^69^.

The studies were categorized into subgroups based on the processed data type, namely text, neuroimage, scale, biomedical, and multidimensional data. We used the STATA MP 17 to conduct subgroup analysis that reported the accuracies vary among subgroups. Forest plots were again used to display the results. We also carried out a sensitivity analysis by excluding one study each time to explore whether the results were driven by a study with an extreme result.

### Supplementary information

Supplementary Material

## Data Availability

Data collected and used in this meta-analysis can be requested from the corresponding author in response to reasonable requests.

## References

[1] Association, A. P. *Diagnostic and Statistical Manual Of Mental Disorders: DSM-5* 5th edn, 271–280 (American Psychiatric Publishing, 2013).

[2] Mota N (2021). Course and predictors of posttraumatic stress disorder in the canadian armed forces: a nationally representative, 16-year follow-up study. Can. J. Psychiatry.

[3] Benjet C (2016). The epidemiology of traumatic event exposure worldwide: results from the World Mental Health Survey Consortium. Psychol. Med..

[4] Yehuda R (2015). Post-traumatic stress disorder. Nat. Rev. Dis. Prim..

[5] Shalev A, Liberzon I, Marmar C (2017). Post-traumatic stress disorder. N. Engl. J. Med..

[6] Song H (2020). Association of stress-related disorders with subsequent neurodegenerative diseases. JAMA Neurol..

[7] Kim YK, Na KS (2018). Application of machine learning classification for structural brain MRI in mood disorders: critical review from a clinical perspective. Prog. Neuro-Psychopharmacol. Biol Psychiatry.

[8] Danmin M (2022). Fusion and model construction of multi-objective measurement of personality traits. J. Air Force Med. Univ..

[9] Jan Z (2021). The role of machine learning in diagnosing bipolar disorder: scoping review. J. Med. Internet Res..

[10] Sawalha J (2022). Detecting presence of PTSD using sentiment analysis from text data. Front. Psychiatry.

[11] Christ NM, Elhai JD, Forbes CN, Gratz KL, Tull MT (2021). A machine learning approach to modeling PTSD and difficulties in emotion regulation. Psychiatry Res..

[12] Held P (2022). Who will respond to intensive PTSD treatment? A machine learning approach to predicting response prior to starting treatment. J. Psychiatr. Res..

[13] Schultebraucks K (2021). Forecasting individual risk for long-term posttraumatic stress disorder in emergency medical settings using biomedical data: a machine learning multicenter cohort study. Neurobiol. Stress.

[14] Kuan PF (2022). Metabolomics analysis of post-traumatic stress disorder symptoms in World Trade Center responders. Transl. Psychiatry.

[15] Khoo LS, Lim MK, Chong CY, McNaney R (2024). Machine learning for multimodal mental health detection: a systematic review of passive sensing approaches. Sens. (Basel).

[16] Dupont T, Kentish-Barnes N, Pochard F, Duchesnay E, Azoulay E (2024). Prediction of post-traumatic stress disorder in family members of ICU patients: a machine learning approach. Intensive Care Med.

[17] Shaw SA, Ward KP, Pillai V, Hinton DE (2019). A group mental health randomized controlled trial for female refugees in Malaysia. Am. J. Orthopsychiatry.

[18] Shiba K (2022). Uncovering heterogeneous associations of disaster-related traumatic experiences with subsequent mental health problems: A machine learning approach. Psychiatry Clin. Neurosci..

[19] Samuelson KW (2022). Mental health and resilience during the coronavirus pandemic: a machine learning approach. J. Clin. Psychol..

[20] Schultebraucks K, Chang BP (2021). The opportunities and challenges of machine learning in the acute care setting for precision prevention of posttraumatic stress sequelae. Exp. Neurol..

[21] Saba T (2022). Machine learning for post-traumatic stress disorder identification utilizing resting-state functional magnetic resonance imaging. Microsc Res. Tech..

[22] Zhu Z (2021). Combining deep learning and graph-theoretic brain features to detect posttraumatic stress disorder at the individual level. Diagnostics.

[23] Yang J (2021). Using deep learning to classify pediatric posttraumatic stress disorder at the individual level. BMC Psychiatry.

[24] Sheynin S (2021). Deep learning model of fMRI connectivity predicts PTSD symptom trajectories in recent trauma survivors. Neuroimage.

[25] Li L (2021). Hippocampal subfield alterations in pediatric patients with post-traumatic stress disorder. Soc. Cogn. Affect Neurosci..

[26] Chen Z (2021). Neural connectome prospectively encodes the risk of post-traumatic stress disorder (PTSD) symptom during the COVID-19 pandemic. Neurobiol. Stress.

[27] Nicholson AA (2020). Classifying heterogeneous presentations of PTSD via the default mode, central executive, and salience networks with machine learning. NeuroImage Clin..

[28] Lanka P (2020). Supervised machine learning for diagnostic classification from large-scale neuroimaging datasets. Brain Imaging Behav..

[29] Kim YW (2020). Riemannian classifier enhances the accuracy of machine-learning-based diagnosis of PTSD using resting EEG. Prog. Neuropsychopharmacol. Biol. Psychiatry.

[30] Harricharan S (2020). PTSD and its dissociative subtype through the lens of the insula: anterior and posterior insula resting-state functional connectivity and its predictive validity using machine learning. Psychophysiology.

[31] Salminen LE (2019). Adaptive identification of cortical and subcortical imaging markers of early life stress and posttraumatic stress disorder. J. Neuroimaging.

[32] Rangaprakash D, Dretsch MN, Katz JS, Denney TS, Deshpande G (2019). Dynamics of segregation and integration in directional brain networks: Illustration in soldiers with PTSD and neurotrauma. Front Neurosci..

[33] Nicholson AA (2019). Machine learning multivariate pattern analysis predicts classification of posttraumatic stress disorder and its dissociative subtype: a multimodal neuroimaging approach. Psychol. Med..

[34] Rangaprakash D (2018). Identifying disease foci from static and dynamic effective connectivity networks: Illustration in soldiers with trauma. Hum. Brain Mapp..

[35] Jin C (2017). Dynamic brain connectivity is a better predictor of PTSD than static connectivity. Hum. Brain Mapp..

[36] Liu F (2015). Characterization of post-traumatic stress disorder using resting-state fMRI with a multi-level parametric classification approach. Brain Topogr..

[37] Nicholson AA (2022). Differential mechanisms of posterior cingulate cortex downregulation and symptom decreases in posttraumatic stress disorder and healthy individuals using real-time fMRI neurofeedback. Brain Behav..

[38] Ramos-Lima LF (2022). Identifying posttraumatic stress disorder staging from clinical and sociodemographic features: a proof-of-concept study using a machine learning approach. Psychiatry Res..

[39] Jiang T (2021). Toward reduced burden in evidence-based assessment of PTSD: a machine learning study. Assessment.

[40] Ge F, Li Y, Yuan M, Zhang J, Zhang W (2020). Identifying predictors of probable posttraumatic stress disorder in children and adolescents with earthquake exposure: A longitudinal study using a machine learning approach. J. Affect Disord..

[41] Leightley D, Williamson V, Darby J, Fear NT (2019). Identifying probable post-traumatic stress disorder: applying supervised machine learning to data from a UK military cohort. J. Ment. Health.

[42] Wshah S, Skalka C, Price M (2019). Predicting posttraumatic stress disorder risk: a machine learning approach. JMIR Ment. Health.

[43] Magoc D, Magoc T, Tomaka J, Morales-Monks S (2016). Automatic identification of firefighters with post-traumatic stress disorder based on demographic characteristics and self-reported alcohol consumption. JUS.

[44] Zafari H, Kosowan L, Zulkernine F, Signer A (2021). Diagnosing post-traumatic stress disorder using electronic medical record data. Health Inform. J..

[45] He Q, Veldkamp BP, Glas CA, de Vries T (2016). Automated assessment of patients’ self-narratives for posttraumatic stress disorder screening using natural language processing and text mining. Assessment.

[46] Lekkas D, Jacobson NC (2021). Using artificial intelligence and longitudinal location data to differentiate persons who develop posttraumatic stress disorder following childhood trauma. Sci. Rep..

[47] Shahid F (2020). Leveraging free-hand sketches for potential screening of PTSD. Proc. ACM Interact. Mob. Wearable Ubiquitous Technol..

[48] Worthington MA, Mandavia A, Richardson-Vejlgaard R (2020). Prospective prediction of PTSD diagnosis in a nationally representative sample using machine learning. BMC Psychiatry.

[49] Tahmasian M (2017). Differentiation chronic post traumatic stress disorder patients from healthy subjects using objective and subjective sleep-related parameters. Neurosci. Lett..

[50] Passos IC (2019). Machine learning and big data analytics in bipolar disorder: a position paper from the International Society for Bipolar Disorders Big Data Task Force. Bipolar Disord..

[51] Ramos-Lima LF, Waikamp V, Antonelli-Salgado T, Passos IC, Freitas LHM (2020). The use of machine learning techniques in trauma-related disorders: a systematic review. J. Psychiatr. Res.

[52] Schultebraucks K, Yadav V, Shalev AY, Bonanno GA, Galatzer-Levy IR (2022). Deep learning-based classification of posttraumatic stress disorder and depression following trauma utilizing visual and auditory markers of arousal and mood. Psychol. Mede.

[53] Sezgin E (2023). Artificial intelligence in healthcare: complementing, not replacing, doctors and healthcare providers. Digit Health.

[54] Ngiam KY, Khor IW (2019). Big data and machine learning algorithms for health-care delivery. Lancet Oncol..

[55] Lee Y (2018). Applications of machine learning algorithms to predict therapeutic outcomes in depression: a meta-analysis and systematic review. J. Affect Disord..

[56] Schultebraucks K, Galatzer-Levy IR (2019). Machine learning for prediction of posttraumatic stress and resilience following trauma: an overview of basic concepts and recent advances. J. Trauma Stress.

[57] Ouyang H (2022). The increase of PTSD in front-line health care workers during the COVID-19 pandemic and the mediating role of risk perception: a one-year follow-up study. Transl. Psychiatry.

[58] Yan W (2024). The impact of isolation on comorbidity of PTSD symptoms and depression: evidence from PTRP-5-6 in China. BMC Public Health.

[59] Liu N (2020). Prevalence and predictors of PTSS during COVID-19 outbreak in China hardest-hit areas: Gender differences matter. Psychiatry Res..

[60] Zhou Y (2021). The prevalence of PTSS under the influence of public health emergencies in last two decades: a systematic review and meta-analysis. Clin. Psychol. Rev..

[61] Jing W, Zhilei S, Weizhi L (2023). Application of machine learning in mental health. J. Nav. Med Univ..

[62] Kelly CJ, Karthikesalingam A, Suleyman M, Corrado G, King D (2019). Key challenges for delivering clinical impact with artificial intelligence. BMC Med..

[63] Al-Hussain G, Shuweihdi F, Alali H, Househ M, Abd-Alrazaq A (2022). The effectiveness of supervised machine learning in screening and diagnosing voice disorders: systematic review and meta-analysis. J. Med Internet Res.

[64] Jathanna N (2021). Diagnostic utility of artificial intelligence for left ventricular scar identification using cardiac magnetic resonance imaging—a systematic review. Cardiovasc Digit Health J..

[65] Eckhardt CM (2023). Unsupervised machine learning methods and emerging applications in healthcare. Knee Surg. Sports Traumatol. Arthrosc..

[66] Page, M. J. et al. The PRISMA 2020 statement: an updated guideline for reporting systematic reviews. *BMJ* **372**, n71-n79 (2021). 10.1136/bmj.n71 PMC8005924 33782057

[67] Wang J (2024). Machine learning methods to discriminate posttraumatic stress disorder: a protocol of systematic review and meta-analysis. Digit Health.

[68] Irwig L, Macaskill P, Berry G, Glasziou P (1998). Bias in meta-analysis detected by a simple, graphical test. Graphical test is itself biased. BMJ.

[69] Egger M, Smith GD, Schneider M, Minder C (1997). Bias in meta-analysis detected by a simple, graphical test. BMJ.

