# Supplementary material for: The application of machine learning techniques in posttraumatic stress disorder: a systematic review and meta-analysis
Source: NPJ Digit Med. 2024 May 9;7:121. doi: 10.1038/s41746-024-01117-5 (PMC11082170; doi:10.1038/s41746-024-01117-5)
Supplement: Supplementary file 1 — Supplementary Material [file 41746_2024_1117_MOESM1_ESM.pdf]

Supplementary Material

related to '**The application of machine learning techniques in posttraumatic stress disorder: a systematic review and meta-analysis**'

Jing Wang [a,d+] , Hui Ouyang [a,d +], Runda Jiao [b+], Suhui Cheng [a,d+], Haiyan Zhang [c] , Zhilei Shang [a,d], Yanpu Jia [a,d],Wenjie Yan [a,d], Lili Wu [a,d *] ,Weizhi Liu [a,d *]

[a] Lab for Post-traumatic Stress Disorder, Faculty of Psychology and Mental Health, Naval Medical University, Shanghai, China, 200433

[b] Graduate School, PLA General Hospital, Beijing, China, 100853

[c] Department of Health Care, The First Affiliated Hospital of Naval Medical University, Shanghai, China 200433

[d] The Emotion & Cognition Lab, Faculty of Psychology and Mental Health, Naval Medical University, Shanghai, China, 200433

+ Jing Wang, Hui Ouyang , Runda Jiao and Suhui Cheng contributed equal to this work.

Correspondence related to this Supplementary Material to wll_88@163.com and 13024141970@163.com.

Table of Contents

Supplementary Table 1. Quality assessment domains. 2
Supplementary Table 2. Quality assessment of the studies. 3
Supplementary Figure 1. Regional trends of publications. 5
Supplementary Figure 2. Galbraith plot of studies using machine learning in posttraumatic stress disorder. 5
Supplementary Figure 3. Sensitivity analysis of studies using machine learning in posttraumatic stress disorder. 6
Supplementary Data 1. Search strategy. 7
Supplementary Table 3. PRISMA 2020 Checklist. 9

Supplementary Table 1. Quality assessment domains.

Feature	Considerations
1. Representativeness of the sample	Was the study truly representative of the target population heterogeneity? If not, was this related to the selected sampling method, insufficient sample size or inclusion/exclusion criteria?
2. Confounding variables	Did the study control for the most relevant confounding variables (age, gender, trauma type)? If so, were covariates assessed using subjective or objective measures?
3. Outcome Assessment	How were outcome measures assessed:<br>A. Independent blind assessment (✓)<br>B. Secure record (e.g., surgical records) (✓)<br>C. Interview not blinded, self-report or medical record<br>D. No description
4. Machine Learning Approach	Was the machine learning algorithm used to analyze data clearly described and appropriate?
5. Performance/Accuracy	Were the following performance metrics included:<br>A. Accuracy<br>B. Sensitivity<br>C. Specificity<br>D. AUC<br>E. PPV/NPV
6. Missing Data	Did the study describe how the authors handled missing data, including if they were inputted or removed?
7. Testing/Validation	Was the test dataset "unseen" in regard to model training? Was the model tested on a hold-out or an external dataset?
8. Class Imbalance	Did the authors address the class imbalance problem? Which method was utilized?
9. Feature Selection and hyperparameter tuning	Did the study describe feature selection or hyperparameter tuning? Which metrics were used?

Supplementary Table 2. Quality assessment of the studies.

First author, year	Sample representativeness	Control confounding	Assessment of the outcome	ML algorithm	Performance metrics	Missing data	Test unseen	Class imbalance	Feature selection/hyperparam
Nicholson,2022	—	—	Y	Y	Y	—	Y	—	Y
Saba,2022	—	—	—	Y	Y	—	Y	—	Y
Chen,2021	—	Y	—	Y	Y	—	Y	Y	Y
Li,2021	—	Y	Y	Y	Y	—	Y	—	Y
Sheynin,2021	—	Y	Y	Y	Y	—	Y	Y	Y
Yang,2021	—	Y	Y	Y	Y	—	Y	Y	Y
Zhu,2021	—	Y	Y	Y	Y	—	Y	—	Y
Harricharan,2020	—	Y	Y	Y	Y	—	—	Y	Y
Kim,2020	—	Y	Y	Y	Y	—	Y	—	Y
Lanka,2020	—	Y	—	Y	Y	—	Y	Y	Y
Nicholson,2020	—	Y	Y	Y	Y	—	Y	Y	—
Nicholson,2019	Y	Y	Y	Y	Y	—	Y	—	Y
Rangaprakash,2019	—	Y	—	Y	Y	—	Y	—	Y
Salminen,2019	—	—	Y	Y	Y	—	Y	Y	Y
Rangaprakash,2018	—	Y	—	Y	Y	—	Y	—	Y
Jin,2017	Y	Y	Y	Y	Y	—	Y	Y	Y
Liu,2015	—	Y	Y	Y	Y	—	Y	—	Y
Ramos-Lima,2022	—	—	Y	Y	Y	Y	Y	Y	Y
Jiang,2021	Y	—	Y	Y	Y	—	Y	—	Y
Ge,2019	Y	—	—	Y	Y	Y	Y	—	Y
Leightley,2019	Y	—	—	—	Y	Y	Y	—	Y
Wshah,2019	—	—	—	Y	Y	Y	Y	—	Y
Magoc,2016	Y	—	—	Y	Y	—	Y	—	—
Shahid,2020	Y	—	—	Y	Y	—	Y	—	Y
Worthington,2020	Y	—	Y	Y	Y	Y	Y	—	Y
Tahmasian,2017	—	Y	Y	Y	Y	—	Y	—	Y
Sawalha,2022	Y	—	—	Y	Y	—	Y	Y	Y

Zafari,2021	Y	—	Y	Y	Y	Y	Y	Y	Y
He,2016	Y	Y	Y	Y	Y	—	—	Y	Y
Lekkas,2021	Y	—	Y	Y	Y	Y	Y	Y	Y
Schultebraucks, 2021	Y	—	Y	Y	Y	Y	Y	Y	Y

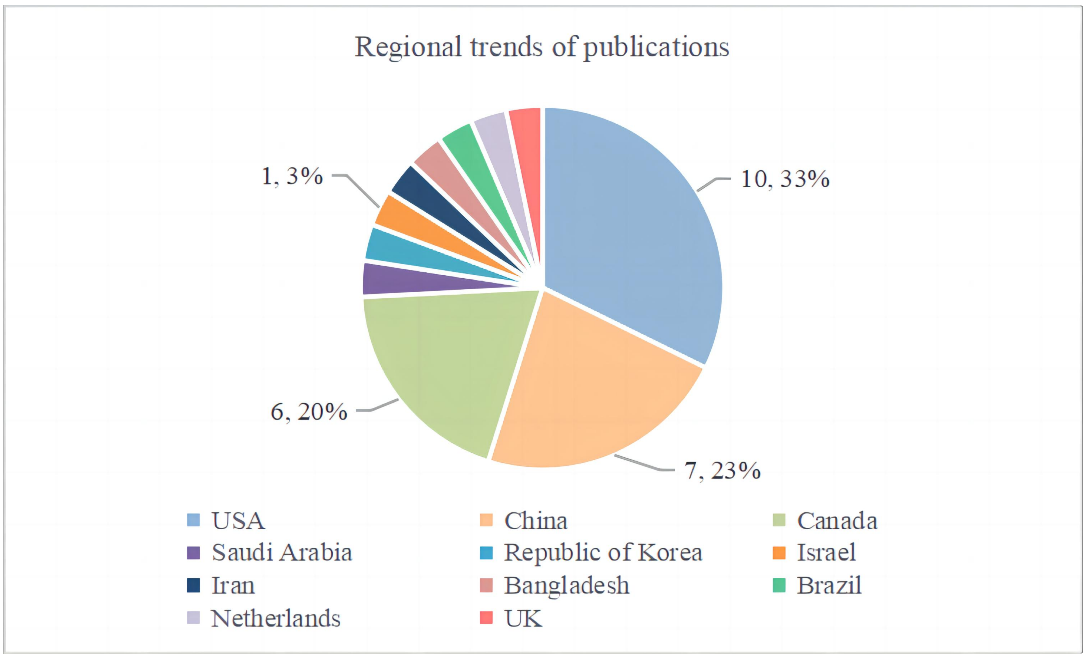

Supplementary Figure 1. Regional trends of publications.

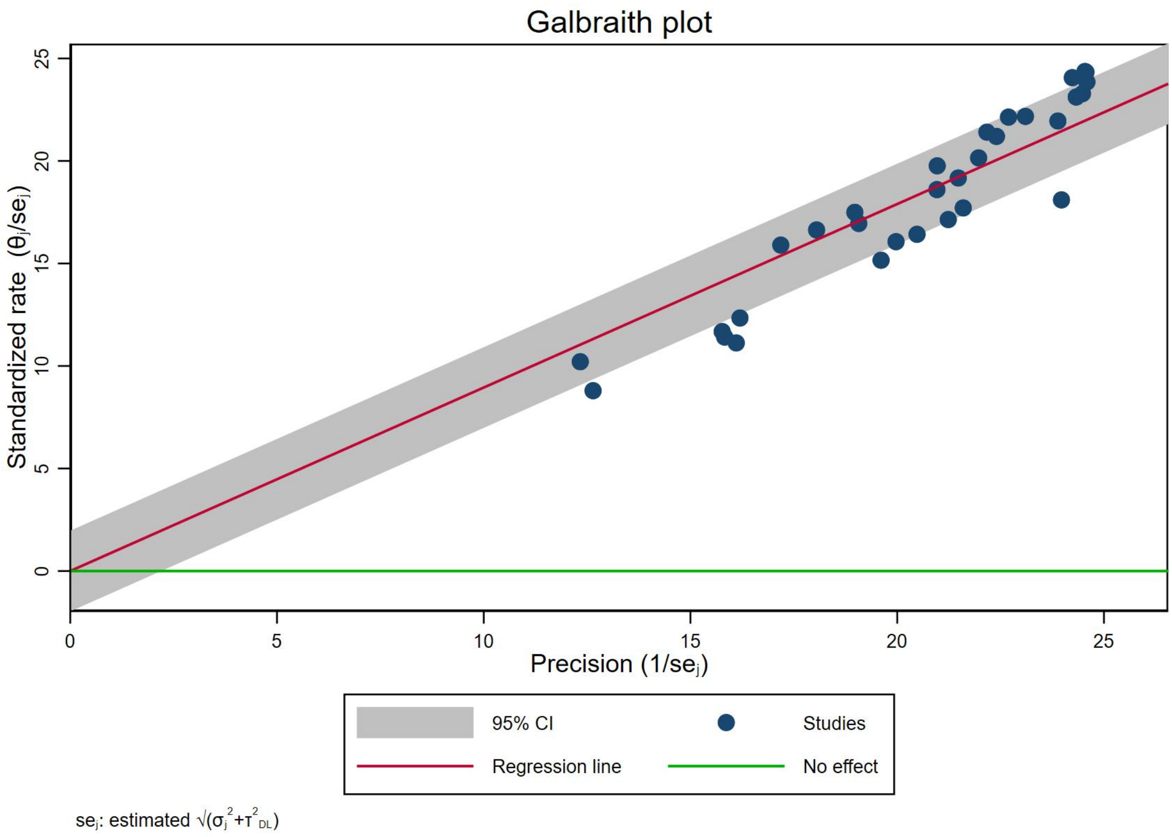

Supplementary Figure 2. Galbraith plot of studies using machine learning in posttraumatic stress disorder.

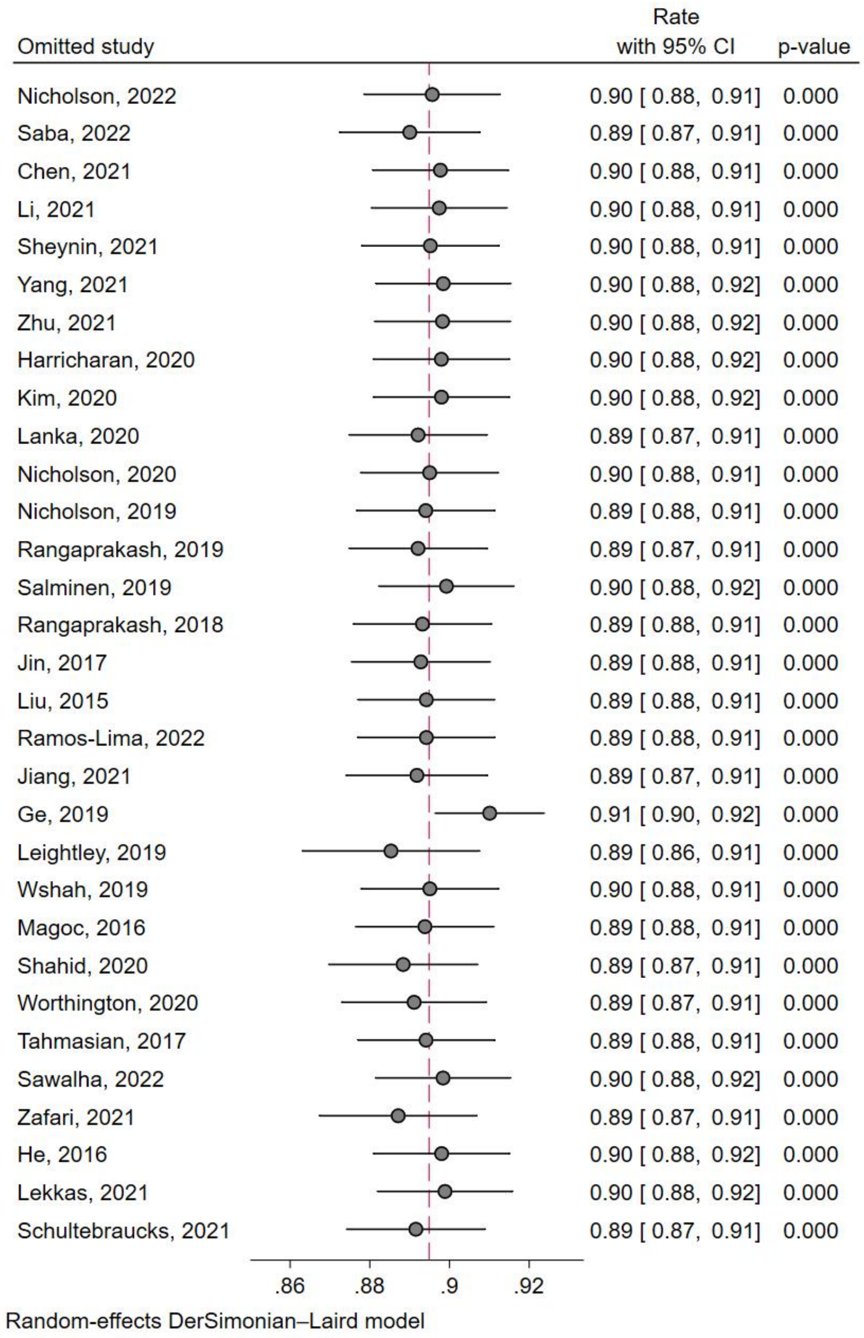

Supplementary Figure 3. Sensitivity analysis of studies using machine learning in posttraumatic stress disorder.

Supplementary Data 1. Search strategy.

(1) Pubmed:
((“Machine Learning”) OR (“Deep Learning”) OR (“Hierarchical Learning”) OR (“Support Vector Machine”) OR (“SVM”) OR (“Reinforcement Learning”) OR (“Natural Language Processing”) OR (“Semi-supervised Learning”) OR (“Gaussian process”) OR (“Cross-validation”) OR (“Cross Validation”) OR (“Regularized Logistic”) OR (“Linear Discriminant Analysis”) OR (“LDA”) OR (“Random Forest”) OR (“Naïve Bayes”) OR (“Naive Bayes”) OR (“Bayesian”) OR (“Least Absolute Shrinkage And Selection Operator”) OR (“LASSO”) OR (“Elastic net”) OR (“RVM”) OR (“Relevance Vector Machine”) OR (“Pattern Recognition”) OR (“Pattern Classification”) OR (“Computational Intelligence*”) OR (“Machine Intelligence”) OR (“Knowledge Representation*”) OR (“Big Data”) OR (“Artificial Intelligence”)) AND ((“PTSD”) OR (“Post-traumatic Stress Disorder*”) OR (“Posttraumatic Stress Disorder*”) OR (“Post Traumatic Stress Disorder*”) OR (“Stress Disorder*, Post Traumatic”) OR (“Stress Disorder*, Posttraumatic”) OR (“Stress Disorder*, Post-traumatic”))

(2) Embase:
Title, abstract or author-specified keywords: ((“Machine Learning”) OR (“Deep Learning”) OR (“Hierarchical Learning”) OR (“Support Vector Machine”) OR (“SVM”) OR (“Reinforcement Learning”) OR (“Natural Language Processing”) OR (“Semi-supervised Learning”) OR (“Gaussian process”) OR (“Cross-validation”) OR (“Cross Validation”) OR (“Regularized Logistic”) OR (“Linear Discriminant Analysis”) OR (“LDA”) OR (“Random Forest”) OR (“Naïve Bayes”) OR (“Naive Bayes”) OR (“Bayesian”) OR (“Least Absolute Shrinkage And Selection Operator”) OR (“LASSO”) OR (“Elastic net”) OR (“RVM”) OR (“Relevance Vector Machine”) OR (“Pattern Recognition”) OR (“Pattern Classification”) OR (“Computational Intelligence*”) OR (“Machine Intelligence”) OR (“Knowledge Representation*”) OR (“Big Data”) OR (“Artificial Intelligence”)) AND ((“PTSD”) OR (“Post-traumatic Stress Disorder*”) OR (“Posttraumatic Stress Disorder*”) OR (“Post Traumatic Stress Disorder*”) OR (“Stress Disorder*, Post Traumatic”) OR (“Stress Disorder*, Posttraumatic”) OR (“Stress Disorder*, Post-traumatic”))

(3) Scopus
TITLE-ABS ((“Machine Learning”) OR (“Deep Learning”) OR (“Hierarchical Learning”) OR (“Supervised Machine Learning”) OR (“Support Vector Machine”) OR (“Support Vector”) OR (“SVM”) OR (“Unsupervised Machine Learning”) OR (“Reinforcement Learning”) OR (“Natural Language Processing”) OR (“Semi-supervised Learning”) OR (“Semi-supervised Machine Learning”) OR (“Gaussian process”) OR (“Cross-validation”) OR (“Cross Validation”) OR (“Regularized Logistic”) OR (“Linear Discriminant Analysis”) OR (“LDA”) OR (“Random Forest”) OR (“Naïve Bayes”) OR (“Naive Bayes”) OR (“Bayesian”) OR (“Least Absolute Shrinkage And Selection Operator”) OR (“LASSO”) OR (“Elastic net”) OR (“RVM”) OR (“Relevance Vector Machine”) OR (“Pattern Recognition”) OR (“Pattern Classification”) OR (“Computational Intelligence*”) OR (“Machine Intelligence”) OR (“Knowledge Representation*”) OR (“Big Data”) OR (“Artificial Intelligence”)) AND ((“PTSD”) OR (“Post-traumatic Stress Disorder*”) OR (“Posttraumatic Stress Disorder*”) OR (“Post Traumatic Stress Disorder*”) OR (“Stress Disorder*, Post Traumatic”) OR (“Stress Disorder*, Posttraumatic”) OR (“Stress Disorder*, Post-traumatic”))

(4) PsycINFO
Title: ((“Machine Learning”) OR (“Deep Learning”) OR (“Hierarchical Learning”) OR (“Supervised Machine Learning”) OR (“Support Vector Machine”) OR (“Support Vector”) OR (“SVM”) OR (“Unsupervised Machine Learning”) OR (“Reinforcement Learning”) OR (“Natural Language Processing”) OR (“Semi-supervised Learning”) OR (“Semi-supervised Machine Learning”) OR (“Gaussian process”) OR (“Cross-validation”) OR (“Cross Validation”) OR (“Regularized Logistic”) OR (“Linear Discriminant Analysis”) OR (“LDA”) OR (“Random Forest”) OR (“Naïve Bayes”) OR (“Naive Bayes”) OR (“Bayesian”) OR (“Least Absolute Shrinkage And Selection Operator”) OR (“LASSO”) OR (“Elastic net”) OR (“RVM”) OR (“Relevance Vector Machine”) OR (“Pattern Recognition”) OR (“Pattern Classification”) OR (“Computational Intelligence*”) OR (“Machine Intelligence”) OR (“Knowledge Representation*”) OR (“Big Data”) OR (“Artificial Intelligence”)) AND ((“PTSD”) OR (“Post-traumatic Stress Disorder*”) OR (“Posttraumatic Stress Disorder*”) OR (“Post Traumatic Stress Disorder*”) OR (“Stress Disorder*, Post Traumatic”) OR (“Stress Disorder*, Posttraumatic”) OR (“Stress Disorder*, Post-traumatic”)) OR Abstract: ((“Machine Learning”) OR (“Deep Learning”) OR (“Hierarchical Learning”) OR (“Supervised Machine Learning”) OR (“Support Vector Machine”) OR (“Support Vector”) OR (“SVM”) OR (“Unsupervised Machine Learning”) OR (“Reinforcement Learning”) OR (“Natural Language Processing”) OR (“Semi-supervised Learning”) OR (“Semi-supervised Machine Learning”) OR (“Gaussian process”) OR (“Cross-validation”) OR (“Cross Validation”) OR (“Regularized Logistic”) OR (“Linear Discriminant Analysis”) OR (“LDA”) OR (“Random Forest”) OR (“Naïve Bayes”) OR (“Naive Bayes”) OR (“Bayesian”) OR (“Least Absolute Shrinkage And Selection Operator”) OR (“LASSO”) OR (“Elastic net”) OR (“RVM”) OR (“Relevance Vector Machine”) OR (“Pattern Recognition”) OR (“Pattern Classification”) OR (“Computational Intelligence*”) OR (“Machine Intelligence”) OR (“Knowledge Representation*”) OR (“Big Data”) OR (“Artificial Intelligence”)) AND ((“PTSD”) OR (“Post-traumatic Stress Disorder*”) OR (“Posttraumatic Stress Disorder*”) OR (“Post Traumatic Stress Disorder*”) OR (“Stress Disorder*, Post Traumatic”) OR (“Stress Disorder*, Posttraumatic”) OR (“Stress Disorder*, Post-traumatic”))

(5) Cochrane Library
((“Machine Learning”) OR (“Deep Learning”) OR (“Hierarchical Learning”) OR (“Supervised Machine Learning”) OR (“Support Vector Machine”) OR (“Support Vector”) OR (“SVM”) OR (“Unsupervised Machine Learning”) OR (“Reinforcement Learning”) OR (“Natural Language Processing”) OR (“Semi-supervised Learning”) OR (“Semi-supervised Machine Learning”) OR (“Gaussian process”) OR (“Cross-validation”) OR (“Cross Validation”) OR (“Regularized Logistic”) OR (“Linear Discriminant Analysis”) OR (“LDA”) OR (“Random Forest”) OR (“Naïve Bayes”) OR (“Naive Bayes”) OR (“Bayesian”) OR (“Least Absolute Shrinkage And Selection Operator”) OR (“LASSO”) OR (“Elastic net”) OR (“RVM”) OR (“Relevance Vector Machine”) OR (“Pattern Recognition”) OR (“Pattern Classification”) OR (“Computational Intelligence*”) OR (“Machine Intelligence”) OR (“Knowledge Representation*”) OR (“Big Data”) OR (“Artificial Intelligence”)) AND ((“PTSD”) OR (“Post-traumatic Stress Disorder*”) OR (“Posttraumatic Stress Disorder*”) OR (“Post Traumatic Stress Disorder*”) OR (“Stress Disorder*, Post Traumatic”) OR (“Stress Disorder*, Posttraumatic”) OR (“Stress Disorder*, Post-traumatic”))

(6) CNKI:
篇关摘：（（机器学习）OR（深度学习）OR（分层学习）OR（监督机器学习）OR（支持矢量机）OR（支持矢量）OR（SVM）OR（无监督机器学习）OR（强化学习）OR（自然语言处理）OR（半监督学习）OR（半监督机器学习）OR（高斯过程）OR（交叉验证）OR（交叉验证）OR（正则化逻辑）OR（线性判别分析）OR（LDA）OR（随机森林）OR（朴素贝叶斯）OR（朴素贝叶斯）OR（朴素）贝叶斯）OR（贝叶斯）OR（最小绝对选择收缩算子）OR（套索）OR（弹性网络）OR（RVM）OR（相关性矢量机）OR（模式识别）OR（模式分类）OR（计算智能*）OR（机器智能）OR（知识表示*）OR（大数据）OR（人工智能））AND 篇关摘：（创伤后应激障碍）

(7) Wanfang:
题名或关键词：（（机器学习）or（深度学习）or（分层学习）or（监督机器学习）or（支持矢量机）or（支持矢量）or（SVM）or（无监督机器学习）or（强化学习）or（自然语言处理）or（半监督学习）or（半监督机器学习）or（高斯过程）or（交叉验证）or（交叉验证）or（正则化逻辑）or（线性判别分析）or（LDA）or（随机森林）or（朴素贝叶斯）or（朴素贝叶斯）or（朴素）贝叶斯）or（贝叶斯）or（最小绝对选择收缩算子）or（套索）or（弹性网络）or（RVM）or（相关性矢量机）or（模式识别）or（模式分类）or（计算智能*）or（机器智能）or（知识表示*）or（大数据）or（人工智能））and 题名或关键词：（创伤后应激障碍）

(8) CSTJ:
篇关摘：（（机器学习）OR（深度学习）OR（分层学习）OR（监督机器学习）OR（支持矢量机）OR（支持矢量）OR（SVM）OR（无监督机器学习）OR（强化学习）OR（自然语言处理）OR（半监督学习）OR（半监督机器学习）OR（高斯过程）OR（交叉验证）OR（交叉验证）OR（正则化逻辑）OR（线性判别分析）OR（LDA）OR（随机森林）OR（朴素贝叶斯）OR（朴素贝叶斯）OR（朴素）贝叶斯）OR（贝叶斯）OR（最小绝对选择收缩算子）OR（套索）OR（弹性网络）OR（RVM）OR（相关性矢量机）OR（模式识别）OR（模式分类）OR（计算智能*）OR（机器智能）OR（知识表示*）OR（大数据）OR（人工智能））AND 篇关摘：（创伤后应激障碍）

Supplementary Table 3. PRISMA 2020 Checklist.

Section and Topic	Item #	Checklist item	Location where item is reported
**TITLE**			
Title	1	Identify the report as a systematic review.	P3
**ABSTRACT**			
Abstract	2	See the PRISMA 2020 for Abstracts checklist.	P3
**INTRODUCTION**			
Rationale	3	Describe the rationale for the review in the context of existing knowledge.	P3-6
Objectives	4	Provide an explicit statement of the objective(s) or question(s) the review addresses.	P6
**METHODS**			
Eligibility criteria	5	Specify the inclusion and exclusion criteria for the review and how studies were grouped for the syntheses.	P20
Information sources	6	Specify all databases, registers, websites, organisations, reference lists and other sources searched or consulted to identify studies. Specify the date when each source was last searched or consulted.	P18-19
Search strategy	7	Present the full search strategies for all databases, registers and websites, including any filters and limits used.	S.P7-8
Selection process	8	Specify the methods used to decide whether a study met the inclusion criteria of the review, including how many reviewers screened each record and each report retrieved, whether they worked independently, and if applicable, details of automation tools used in the process.	P20-21
Data collection process	9	Specify the methods used to collect data from reports, including how many reviewers collected data from each report, whether they worked independently, any processes for obtaining or confirming data from study investigators, and if applicable, details of automation tools used in the process.	P21-22
Data items	10a	List and define all outcomes for which data were sought. Specify whether all results that were compatible with each outcome domain in each study were sought (e.g. for all measures, time points, analyses), and if not, the methods used to decide which results to collect.	P21-22
	10b	List and define all other variables for which data were sought (e.g. participant and intervention characteristics, funding sources). Describe any assumptions made about any missing or unclear information.	P21-22
Study risk of bias assessment	11	Specify the methods used to assess risk of bias in the included studies, including details of the tool(s) used, how many reviewers assessed each study and whether they worked independently, and if applicable, details of automation tools used in the process.	P22
Effect measures	12	Specify for each outcome the effect measure(s) (e.g. risk ratio, mean difference) used in the synthesis or presentation of results.	P23
Synthesis methods	13a	Describe the processes used to decide which studies were eligible for each synthesis (e.g. tabulating the study intervention characteristics and comparing against the planned groups for each synthesis (item #5)).	P20-21
	13b	Describe any methods required to prepare the data for presentation or synthesis, such as handling of missing summary statistics, or data conversions.	P23
	13c	Describe any methods used to tabulate or visually display results of individual studies and syntheses.	P23
	13d	Describe any methods used to synthesize results and provide a rationale for the choice(s). If meta-analysis was performed, describe the model(s), method(s) to identify the presence and extent of statistical heterogeneity, and software package(s) used.	P23
	13e	Describe any methods used to explore possible causes of heterogeneity among study results (e.g. subgroup analysis, meta-regression).	P23
	13f	Describe any sensitivity analyses conducted to assess robustness of the synthesized results.	P23
Reporting bias assessment	14	Describe any methods used to assess risk of bias due to missing results in a synthesis (arising from reporting biases).	P23

Section and Topic	Item #	Checklist item	Location where item is reported
Certainty assessment	15	Describe any methods used to assess certainty (or confidence) in the body of evidence for an outcome.	P22
**RESULTS**			
Study selection	16a	Describe the results of the search and selection process, from the number of records identified in the search to the number of studies included in the review, ideally using a flow diagram.	P6-7
	16b	Cite studies that might appear to meet the inclusion criteria, but which were excluded, and explain why they were excluded.	P6
Study characteristics	17	Cite each included study and present its characteristics.	P7
Risk of bias in studies	18	Present assessments of risk of bias for each included study.	S.P3-4
Results of individual studies	19	For all outcomes, present, for each study: (a) summary statistics for each group (where appropriate) and (b) an effect estimate and its precision (e.g. confidence/credible interval), ideally using structured tables or plots.	Table 1
Results of syntheses	20a	For each synthesis, briefly summarise the characteristics and risk of bias among contributing studies.	P9-11
	20b	Present results of all statistical syntheses conducted. If meta-analysis was done, present for each the summary estimate and its precision (e.g. confidence/credible interval) and measures of statistical heterogeneity. If comparing groups, describe the direction of the effect.	P8-9
	20c	Present results of all investigations of possible causes of heterogeneity among study results.	P9-10
	20d	Present results of all sensitivity analyses conducted to assess the robustness of the synthesized results.	P9
Reporting biases	21	Present assessments of risk of bias due to missing results (arising from reporting biases) for each synthesis assessed.	P9
Certainty of evidence	22	Present assessments of certainty (or confidence) in the body of evidence for each outcome assessed.	P9
**DISCUSSION**			
Discussion	23a	Provide a general interpretation of the results in the context of other evidence.	P11-12
	23b	Discuss any limitations of the evidence included in the review.	P12-16
	23c	Discuss any limitations of the review processes used.	P12-16
	23d	Discuss implications of the results for practice, policy, and future research.	P14-18
**OTHER INFORMATION**			
Registration and protocol	24a	Provide registration information for the review, including register name and registration number, or state that the review was not registered.	P18
	24b	Indicate where the review protocol can be accessed, or state that a protocol was not prepared.	P18
	24c	Describe and explain any amendments to information provided at registration or in the protocol.	P18
Support	25	Describe sources of financial or non-financial support for the review, and the role of the funders or sponsors in the review.	P24
Competing interests	26	Declare any competing interests of review authors.	P24
Availability of data, code and other materials	27	Report which of the following are publicly available and where they can be found: template data collection forms; data extracted from included studies; data used for all analyses; analytic code; any other materials used in the review.	P24
